# Framing the implementation process of a social innovation project for people with severe mental disorders in chile

**DOI:** 10.1192/j.eurpsy.2021.1250

**Published:** 2021-08-13

**Authors:** M. Solis-Soto, G. Reginatto, R. Alvarado, T. Arratia, M. Burrone

**Affiliations:** Instituto De Ciencias De La Salud, Universidad de O’Higgins, Rancagua, Chile

**Keywords:** implementation, Socio-labour inclusion, work cooperatives

## Abstract

**Introduction:**

There are important challenges for the effective inclusion in society of people with severe mental disorders (SMD). The POTENCIA^T^ intervention supports the formation of work cooperatives for the socio-labour inclusion of people with SMD working together with women in vulnerable situations and older adults.

**Objectives:**

To analyse the implementation process of the first phase of POTENCIA^T^

**Methods:**

According to Nilsen, six components were analysed: implementation object, implementation activities, implementation actors, users, inner context and outer context. A qualitative study was performed during the first phase of the project (12-months). Participant observation and in-depth interviews with key actors, users and fieldwork team were conducted, as well as bibliographic analysis of field notes, meeting minutes and audios/videos of socialization activities. Ethical issues were considered.

**Results:**

The implementation object was positively valued as a response to perceived needs such as users involvement in a participatory approach. Shared decision-making process was emphasized. Implementation activities were accompanied by local teams and were adapted to participants needs and context characteristics (e.g. social distancing). Most users showed high expectations related to the solidarity economic model as well as physical and economic autonomy, which work as a key factor for commitment and retention in the project. A good working environment was recognized and a space for personal fulfillment. Some barriers were recognized, including cultural stigma, auto-stigma, and recruitment in pandemic context.

**Conclusions:**

Early and proper addressing of factors that may positively and negatively affect the implementation process is mandatory to achieve the effective inclusion of people with SMD.

